# Exosomes isolated from the plasma of remote ischemic conditioning rats improved cardiac function and angiogenesis after myocardial infarction through targeting Hsp70

**DOI:** 10.18632/aging.102837

**Published:** 2020-02-18

**Authors:** Qin Chen, Minghan Huang, Jiayi Wu, Qiong Jiang, Xingchun Zheng

**Affiliations:** 1 Department of Cardiology, Fujian Medical University Union Hospital, Fuzhou, Fujian 350001, P.R. China; 2 Fujian Institute of Coronary Artery Disease, Fuzhou, Fujian 350001, P.R. China; 3 Fujian Heart Medical Center, Fuzhou, Fujian 350001, P.R. China; 4 The Second Affiliated Hospital of Fujian Traditional Chinese Medical University, Fuzhou, Fujian 350003, P.R. China

**Keywords:** exosomes, remote ischemic conditioning, myocardial infarction, Hsp70

## Abstract

Remote ischemic conditioning (RIC) is a promising therapeutic strategy to protect heart against ischemic-reperfusion injury. Exosomes have been proved to be an important regulator in many pathological processes. Whether the exosomes derived from RIC could improve cardiac remodeling and function after myocardial infarction (MI) has not been reported. MI animal model was established by ligating the left coronary artery. The bilateral hindlimbs of rats were subjected to RIC treatment using tourniquets. Exosomes were isolated from the plasma of RIC rats and identified by transmission electron microscope. The proliferation, migration, and apoptosis of endothelial cells were measured by CCK8, traswell, and flow cytometry. Western blotting, and qRT-PCR were applied to measure the expression of angiogenesis-related molecules, and immunohistochemistry staining was used to observe the expression of vWF. RIC and RIC exosomes remarkably facilitated cardiac function, cardiac cell remodeling, and angiogenesis. RIC exosomes markedly increased the cell ratio in the G1 phase, cell migration, cell proliferation, tube formation, and inhibited cell apoptosis through Hsp70. The expression of eNOS, iNOS, HIF-1α, Ang-1, and VEGF was markedly increased by RIC exosomes. RIC exosomes significantly improved cardiac function, cardiac remodeling, and angiogenesis after MI, and they accelerated angiogenesis through increasing the levels of angiogenesis-related molecules.

## INTRODUCTION

Cardiac dysfunction after myocardial infarction (MI) is one of the major causes of morbidity and mortality in the world [1]. Although significant improvement of therapeutic strategies has elevated the survival of MI patients, treatment such as timely reperfusion still commonly causes ischemia-reperfusion injury, which could result in a systemic inflammatory response and even organ dysfunction [2]. The temporary ischemia-reperfusion event in the leg or distant organ is known as remote ischemic conditioning (RIC), which has been believed to be a potential method to relieve ischemia-reperfusion injury [3]. Several reports demonstrated that treatment with RIC before MI (Pre-RIC) or after the onset of reperfusion (Post-RIC) could exert a cardioprotective effect on MI [4].

Exosomes are known as biological nanovesicles, characterized by 40–120 nm in diameter [5]. Exosomes are secreted from different kinds of cells, and able to transport microRNA and messenger RNA to relative receptors [6]. Exosomes have been confirmed to be a vital regulator in the process of neovascularization, cell proliferation, and apoptosis [7]. Meanwhile, the exosomes isolated from different types of stem cells have presented a cardioprotective effect on ischemia-reperfusion injury [8]. However, whether the exosomes isolated from the plasma of RIC rats could exert protective influence on MI has not been investigated.

Heat-shock proteins can be stimulated by different kinds of factors including ischemia, glucose deprivation, heat shock, and hypoxia [9]. The 70-kDa Hsp (Hsp70) are investigated extensively, and they have been proved to be an important regulator in protein synthesis, folding, assembly, and trafficking between different cells [10]. It has been reported that Hsp70 was reported to be closely linked with angiogenesis [11]. Meanwhile, Hsp70 acted a key role in regulating the angiogenic effects caused by interleukin-5 (IL-5) through endothelial nitric oxide synthase (eNOS) signaling pathway [12], which has been considered to be a producer of nitric oxide (NO), a type of vasoactive substances, during neoangiogenesis via vascular endothelial growth factor (VEGF). However, whether RIC exosomes could improve cardiac remodeling and angiogenesis after MI through targeting Hsp70 or other targets remains unknown.

Endothelial cells act a vital role in regulating vascular function through releasing vasoactive substances including VEGF, NO, angiopoietin-1 (Ang-1), and hypoxia-inducible factor 1 α (HIF-1 α) [13, 14]. The dysfunction of endothelial cells is closely linked with bad clinical outcomes after ischemia-reperfusion injury [15]. In the present study, cardiac microvascular endothelial cells (CMVECs) were selected to measure the influence of RIC exosomes on angiogenesis and endothelial cell function. Meanwhile, we investigated the effect of RIC exosomes on cardiac remodeling and angiogenesis based on the MI animal model. This study unfolds the potential therapeutic function of RIC exosomes on MI damage and may provide a novel insight into the prevention and treatment of MI.

In the present study, we identified the protective effect of RIC on cardiac remodeling and function. Meanwhile, we firstly proved that the exosomes derived from RIC rats could also significantly improve the cardiac remodeling and angiogenesis after MI. Hsp70 and other angiogenesis related molecules including eNOS, iNOS, HIF-1α, Ang-1, and VEGF might be the regulatory molecules exosomes mediating angiogenesis and improvement of MI damage. This study provides a novel therapeutic strategy for the improvement of MI damage.

## RESULTS

### RIC remarkably promoted cardiac cell remodeling and angiogenesis after myocardial infarction

**Figure 1:**
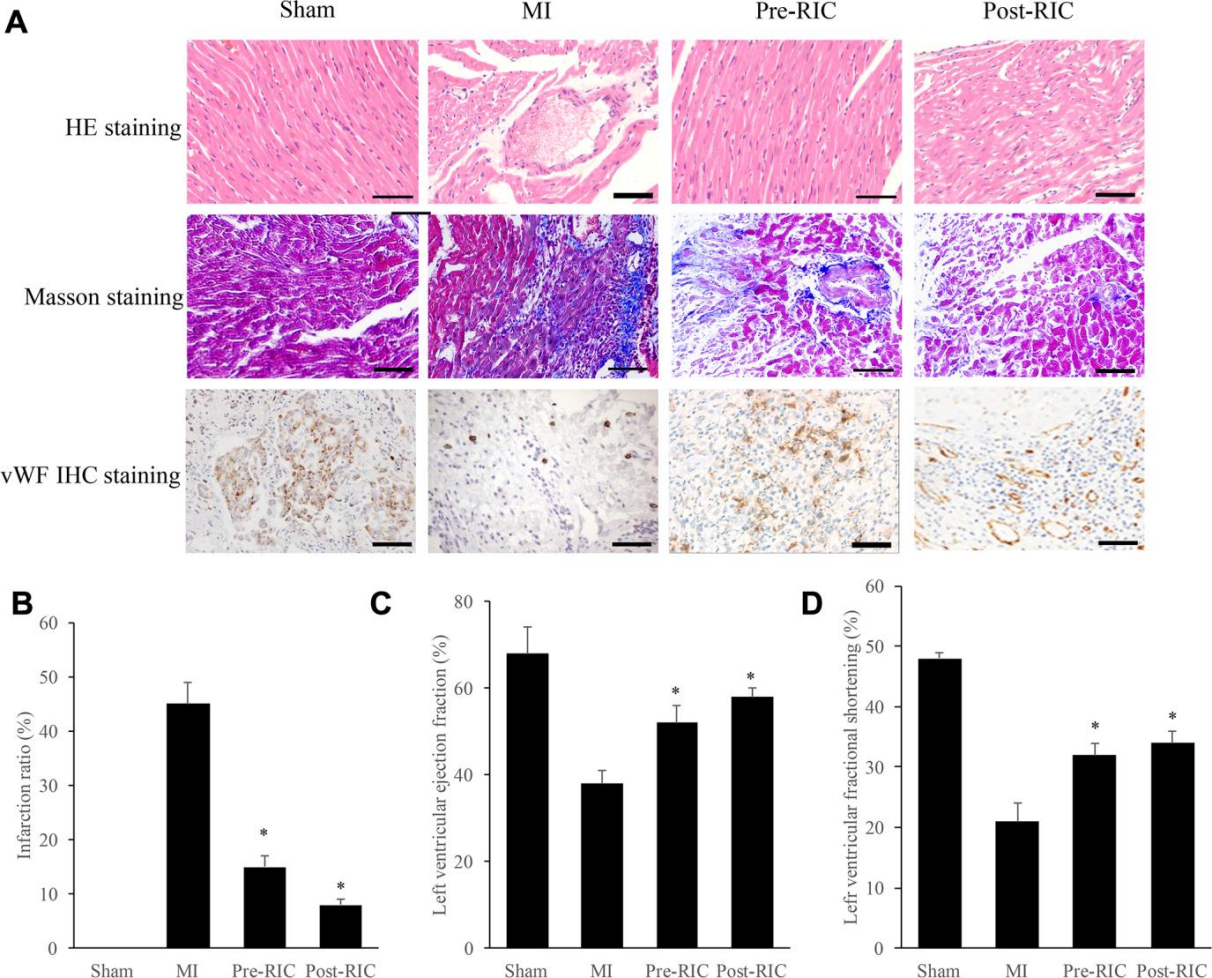
**RIC remarkably promoted cardiac remodeling and angiogenesis after myocardial infarction.** (**A**) Histopathological analysis of heart tissues by HE, Masson, vWF IHC staining, respectively (scale bar= 100 *μ*m); (**B**) RIC treatment significantly decreased the infarction ratio; (**C**) RIC treatment significantly increased left ventricular ejection fraction; (**D**) RIC treatment significantly increased left ventricular fractional shortening. Data were shown as the mean ± SD (n = 5/each group), * P<0.05 compared with the group MI.

Morphological changes were measured with HE and Masson staining (Figure 1A). In the group MI, the arrangement of cardiomyocytes was disordered, and the gap between cells was enlarged remarkably, but treatment with RIC significantly improved this morphological change (Figure 1A). Meanwhile, Masson staining was applied to evaluate myocardial fibrosis, which could accelerate cardiac dysfunction. Remarkable interstitial collagen deposition was found in MI rats, but therapy with RIC markedly decreased the density of collagen (Figure 1A). The results of infarction ratio analyzed by image J were in line with the Masson staining view. The infarction ratio was remarkably decreased after treatment with RIC (Figure 1B). Moreover, the angiogenesis in the myocardial tissues was investigated by measuring the expression of vWF. Newly formed small blood vessels could be observed in the group Pre-RIC and Post-RIC (Figure 1A). Similar findings were observed in terms of left ventricular fractional shortening (LVFS) and left ventricular ejection fraction (LVEF). In group MI, both LVFS and LVEF were significantly decreased compared with group sham. However, either pre-treatment or post-treatment with RIC could markedly increase the levels of LVEF and LVFS compared with group MI (Figure 1C and 1D).

### Isolation of exosomes from RIC rats

**Figure 2:**
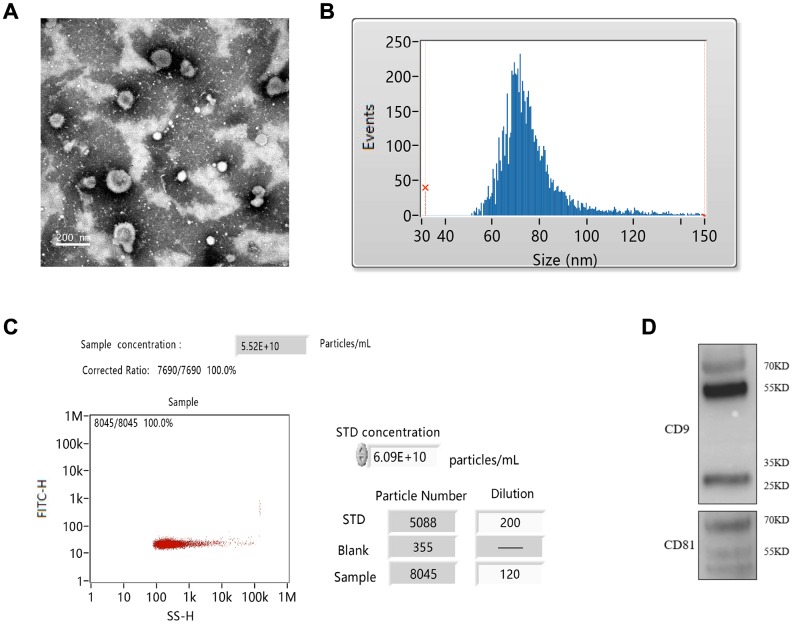
**Isolation of exosomes from RIC rats.** (**A**) Exosomes identification by TEM; (**B**) Measurement of exosomes particle size; (**C**) Measurement of exosomes concentration by flow cytometry; (**D**) Identification of CD9 and CD81 by western blotting.

The exosomes were isolated from the plasma of RIC rats. The isolated exosomes were spherical shape and uniform size identified by TEM (Figure 2A). The average particle size was 76.23 nm, and 30-150 nm of exosomes accounted for 98.37% of the total particles (Figure 2B). The concentration of prepared exosomes was 5.25E+10 particles/mL measured by flow cytometry (Figure 2C). Specific antibodies, CD9 and CD81 were also investigated by western blotting (Figure 2D).

### Exosomes from RIC rats significantly promoted cardiac remodeling and angiogenesis after myocardial infarction

**Figure 3:**
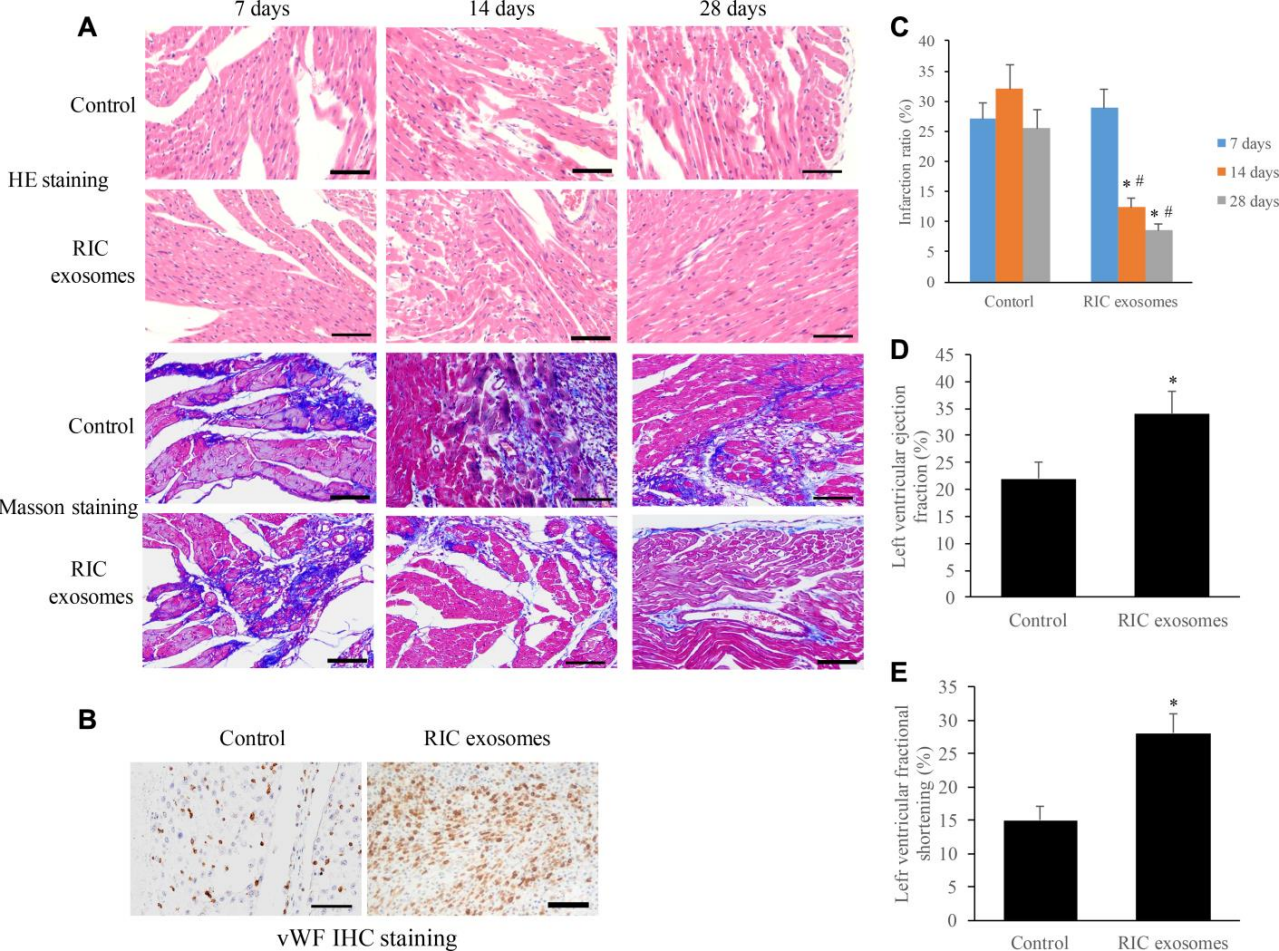
**Exosomes from RIC rats significantly promoted cardiac remodeling and angiogenesis after myocardial infarction.** (**A**) Histopathological analysis of heart tissues by HE and Masson (scale bar= 100 *μ*m); (**B**) Investigation of angiogenesis after RIC exosomes treatment by vWf IHC staining (scale bar= 100 *μ*m); (**C**) RIC exosomes treatment significantly decreased the infarction ratio; (**D**) RIC treatment significantly elevated left ventricular ejection fraction; (**E**) RIC treatment significantly promoted left ventricular fractional shortening. Data were shown as the mean ± SD (n = 3/each group), * P<0.05 compared with respective time point in the control group, # P<0.05 compared with 7 days’ time point in the group RIC exosomes.

We examined the morphological changes of myocardial tissues at different time points after treatment with RIC exosomes or normal saline. The increase of myocyte cross-sectional area and loose arrangement of cells were found in the group control, and no significant improvement was observed after 28 days (Figure 3A). However, after 28 days’ treatment with RIC exosomes, cardiomyocytes arranged regularly and the intercellular space narrowed remarkably (Figure 3A). The results of Masson staining indicated that the collagen deposition was significantly decreased in the group RIC exosomes at the 14^th^ and 28^th^ days compared with group control (Figure 3A). The results of Masson were in line with the analysis of infarction ratio suggesting that the infarction ratio was remarkably suppressed after treatment with RIC exosomes (Figure 3C). Meanwhile, vWF IHC staining indicated that a significant increase of small blood vessels was observed after treatment with RIC exosomes on the 28^th^ day (Figure 3B). Moreover, therapy with RIC exosomes markedly increased the levels of LVEF and LVFS compared with control (Figure 3D and 3E).

### Exosomes from RIC markedly improved the cell viability and angiogenesis through promoting Hsp70

**Figure 4:**
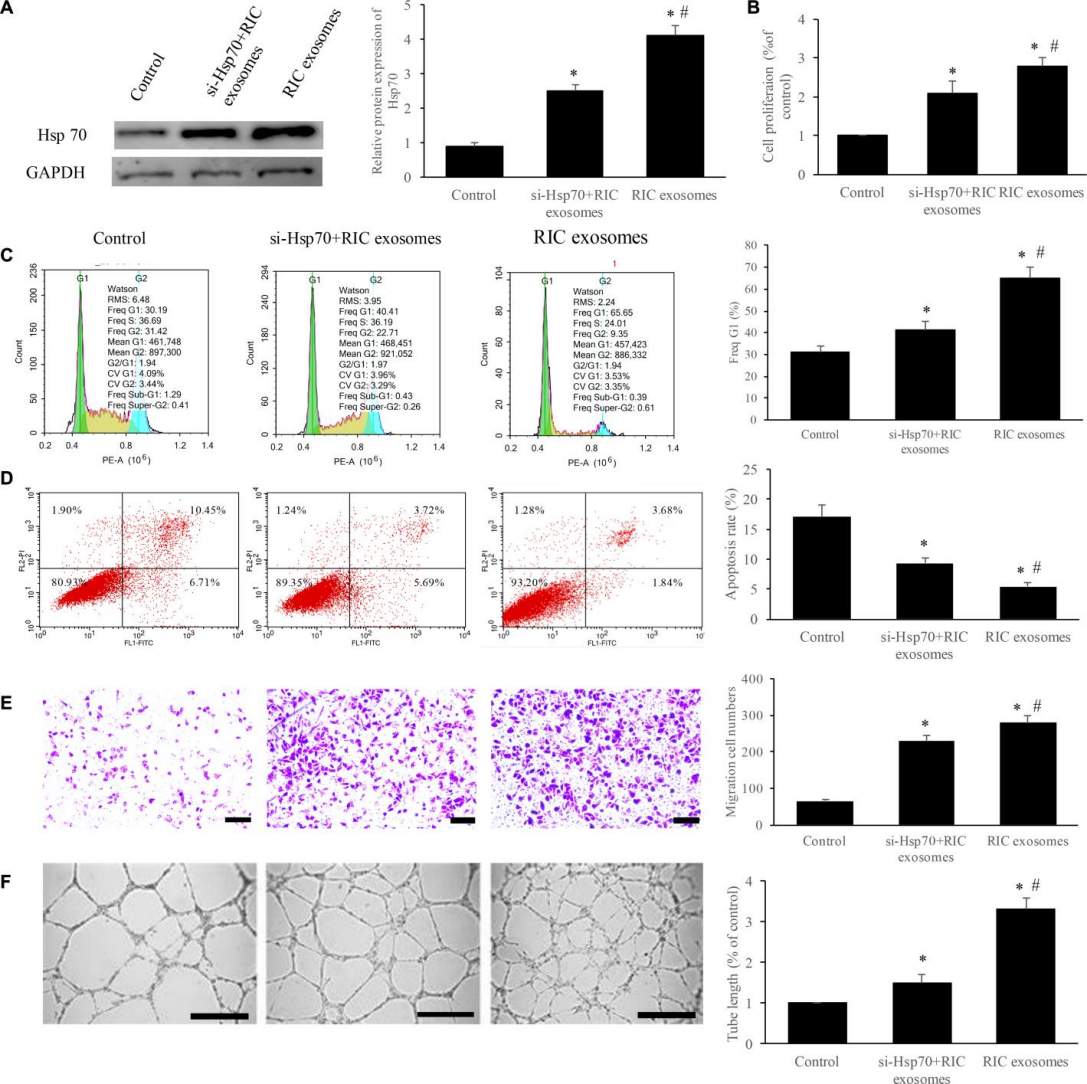
**Exosomes from RIC markedly improved the cell viability and angiogenesis through promoting Hsp70.** (**A**) RIC exosomes remarkably elevated the expression of Hsp70; (**B**) RIC exosomes significantly accelerated cell proliferation; (**C**) RIC exosomes remarkably increased the ratio of cell in G1 stage; (**D**) RIC exosomes significantly inhibited cell apoptosis; (**E**) RIC exosomes remarkably promoted cell migration (scale bar= 500 *μ*m); (**F**) RIC exosomes significantly increased tube formation (scale bar= 500 *μ*m). * P<0.05 compared with the control group. # P<0.05 compared with group si-Hsp70+ RIC exosomes.

To further unfold the potential mechanism, we measured the expression of Hsp70 after treatment with RIC exosomes, and si-Hsp70 was used to knockdown the level of Hsp70. We found that significant increase of Hsp70 expression was achieved after incubation with RIC exosomes, but simultaneous treatment with RIC exosomes and si-Hsp70 remarkably suppressed Hsp70 compared with group RIC exosomes (Figure 4A). Besides, the cell cycle, apoptosis, cell migration, and tube formation were investigated. RIC exosomes markedly increased the cell percentage in the G1 stage, cell migration, cell proliferation, tube formation, and inhibited cell apoptosis (Figure 4B–4F). However, simultaneous treatment with RIC exosomes and si-Hsp70 remarkably reversed the influence caused by RIC exosomes indicating that Hsp70 should be a functioning target of RIC exosomes.

### The expression increase of angiogenesis-related molecules after incubation with exosomes through targeting Hsp70

**Figure 5:**
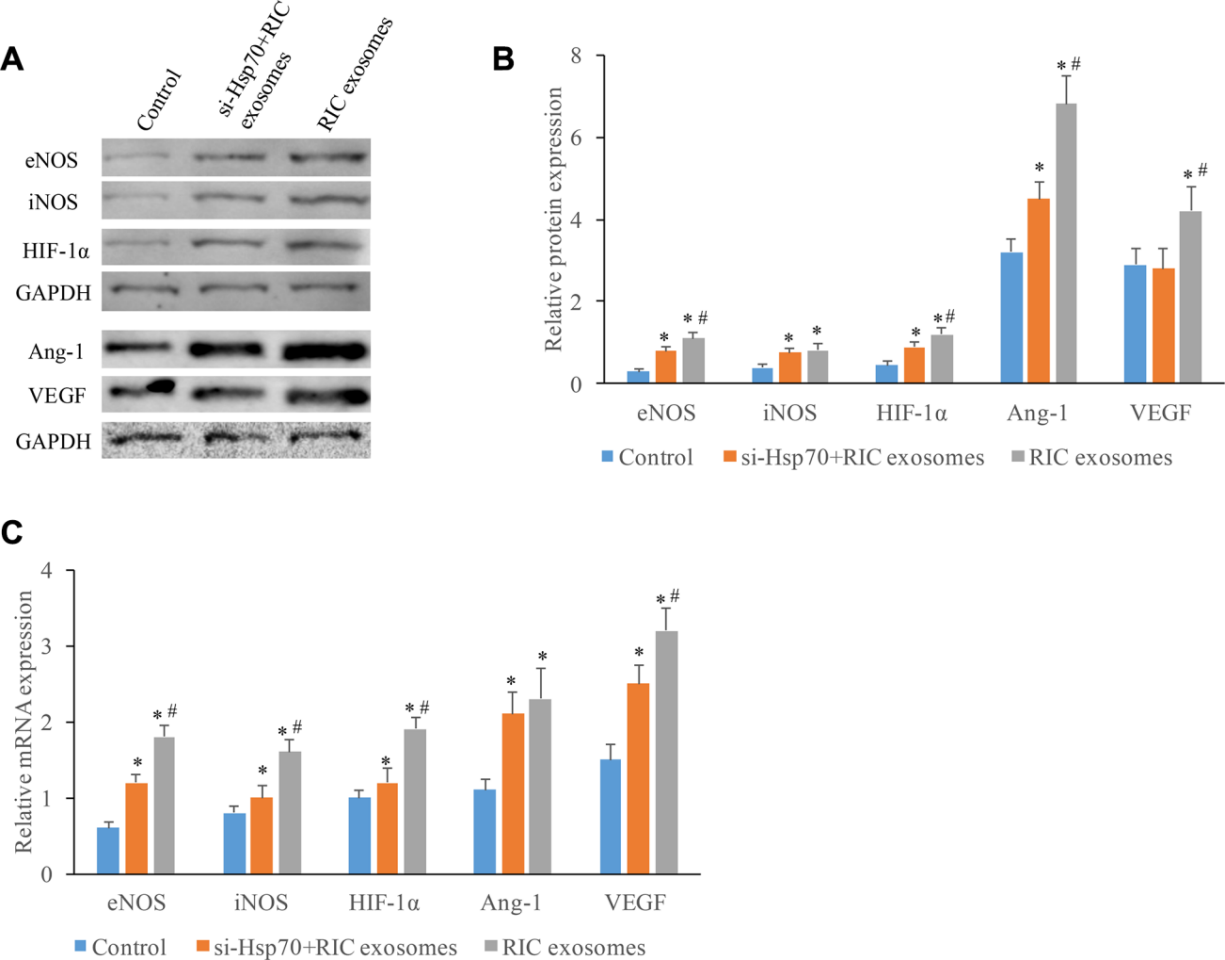
**The expression increase of angiogenesis-related molecules after incubation with exosomes through targeting Hsp70.** (**A**) Western analysis of angiogenesis-related molecule after treatment with si-Hsp70 and RIC exosomes; (**B**) Protein quantitative analysis of angiogenesis-related molecule after treatment with si-Hsp70 and RIC exosomes; (**C**) mRNA expression of angiogenesis-related molecule after treatment with si-Hsp70 and RIC exosomes. * P<0.05 compared with the control group. # P<0.05 compared with group si-Hsp70+RIC exosomes.

Several other molecules linked with angiogenesis were also investigated. Significant protein expression promotion of eNOS, iNOS, HIF-1α, Ang-1, and VEGF was achieved by RIC exosomes (Figure 5A, 5B). However, co-incubation with si-Hsp70 could reverse these trends, and similar results in terms of the influence of RIC exosomes and si-Hsp70 on mRNA expression of eNOS, iNOS, HIF-1α, Ang-1, and VEGF were observed (Figure 5C). These findings indicated that RIC exosomes might improve cardiac remodeling and angiogenesis by targeting Hsp70.

## DISCUSSION

The mortality and morbidity of MI remain high despite timely reperfusion [16], and RIC has been proved to a promising treatment to decrease myocardial infarct size, improve cardiac function, and reduce the risk of heart failure in animal models [17, 18]. LVEF is the ratio between output per stroke and the volume of end diastolic ventricle, LVFS is an index parameter of left ventricular systolic function. They are all important indicators to judge cardiac function. Any disease that affects myocardial function damage could change LVEF and LVFS. Our findings in this study demonstrated that RIC markedly improved cardiac cell remodeling, decreased infarction ratio, promoted angiogenesis, increased LVEF and LVFS (Figure 1), which are in line with previous reports. The protective effect of RIC on cardiac cells has been confirmed to be related to its improvement to the antioxidant system by promoting the levels of catalase, superoxide dismutase, and GSH peroxidase [19, 20]. However, a single-blind, randomized controlled trial concluded that RIC did not improve clinical outcomes (hospitalization or cardiac death for heart failure) at 12 months in patients with ST-elevation acute myocardial infarction undergoing primary percutaneous coronary intervention [21]. The simplicity of the animal MI model and the difference between animals and humans may account for the different outcomes of RIC on animals and humans.

Meanwhile, RIC may also exert a protective effect on target organs through promoting the secretion of microRNAs, circular RNA, transcription factors, chemokines, and cytokines, which are encased in the form of exosomes. However, the influence of exosomes isolated from RIC plasma on MI has not been reported. We proved that the RIC exosomes significantly improved cardiac cell remodeling, inhibited collagen deposition, decreased infarction ratio, facilitate angiogenesis, increased LVEF and LVFS (Figure 3). Therefore, the protective effect of RIC on MI might be contributed by exosomes. However, the specific components in the exosomes contributing to the improvement of heart function after MI needs to be investigated further.

We also found that RIC exosomes remarkably promoted the proliferation and migration of endothelial cells, increased the cell percentage in the G1 stage, suppressed apoptosis and facilitate tube formation (Figure 4). Moreover, several microRNAs including microRNA-130a [22]and microRNA-10a [23] have presented a similar effect on the viability of endothelial cells. Therefore, the expression of these microRNAs in the RIC exosomes and whether RIC exosomes protect the heart function through these microRNAs need further study.

Vicencio JM et al has demonstrated that exosomal HSP70 could present cardioprotective effect by stimulatingTLR4 signaling, activating ERK1/2 and p38MAPK, and then phosphorylating HSP27 [24]. Our findings that both RIC and RIC exosomes could remarkably improve the cardiac function by regulating Hsp70 are consistent with the results of Vicencio JM et al. Meanwhile, we investigate the the influence of RIC exosomes on cell viability of CMVECs, angiogenesis, and angiogenesis related molecules. Angiogenesis is beneficial to alleviate functional and structural damage caused by ischemia-reperfusion, and further facilitate cardiac recovery [25]. Therefore, treatment promoting myocardial angiogenesis has been viewed as a potential therapeutic strategy for patients with MI. Significant increase of vWF expression in vivo and tube branches in vitro after RIC exosomes treatment indicating that RIC exosomes could promote myocardial angiogenesis. iNOS and eNOS have been shown to protect tissues from ischemia-reperfusion injury through suppressing apoptosis and increasing capillary density after MI. Meanwhile, co-expression Ang1 and VEGF can improve cardiac function in a porcine MI model through promoting cardiomyocyte proliferation and angiogenesis, inhibiting apoptosis [26]. HIF-1α plays an important role in improving vascular function after MI through regulating interleukin-10 (IL-10) [20]. Significant expression increase of eNOS, iNOS, HIF-1α, Ang-1, and VEGF caused by RIC exosomes and inhibition of them after si-Hsp70 indicate that RIC exosomes may improve angiogenesis and cardiac remodeling through Hsp70 targeting these molecules, but the specific mechanism needs to be investigated further.

In summary, we identified the improvement of cardiac function and angiogenesis by Pre-RIC and Post-RIC treatment after MI. Meanwhile, the RIC exosomes remarkably accelerated the recovery of cardiac function, improved cardiac remodeling, and promoted new small vessel formation. We firstly demonstrated that RIC exosomes could markedly promote the cell ratio in G1 stage, cell proliferation, cell migration, new tube formation, and inhibited cell apoptosis in vitro through si-Hsp70. Besides, RIC exosomes might accelerate angiogenesis by promoting the expression of angiogenesis-related molecules. This study unfolds the possible therapeutic mechanism of RIC exosomes on MI damage and provides a new thought on the prevention and treatment of MI.

## MATERIALS AND METHODS

### Isolation, preparation, and identification of RIC exosomes

Plasma of RIC rats was used to isolate exosomes. The plasma was centrifuged at 2000 g for 20 min at 4 °C. After careful removal of supernatant, the plasma was centrifuged again at 12,000 g for 40 min at 4 °C to remove bigger vesicles. Then, supernatant was filtered using a 0.45 μm membrane. The filtered solution was centrifuged at 110,000 g for 70 min at 4 °C, after removing supernatant cold PBS was used for re-suspension. The mixed solution was centrifuged again at 120,000 g for 70 min at 4 °C. Remove the supernatant and re-suspend solution with cold PBS. The particle size and concentration were analyzed using Flow NanoAnalyzer (NanoFCM, Nottingham, UK). The specific biomarkers (CD81 and CD9) were measured using western blot.

### Cell culture

We purchased CMVECs from Life line cell technology (Oceanside, CA, US). The hypoxia model was established with a specific incubator (5% CO_2_, 1% O_2_, and 94% N_2_). CMVECs were culture in this incubator for 12 h. The prepared exosomes were diluted 10 times with PBS buffer and then applied for cell culture. The cells were incubated with RIC exosomes + siRNA of Hsp70 (si-Hsp70) or RIC exosomes for an additional 24 h. The treated cells were applied to other experiments. The si-Hsp70 was designed and synthesized by GenePharma Co., Ltd (Shanghai, China). The specific information of siRNA was listed as follows: scrambled siRNA:5-CCUCGUGCCGUUCCAUCAGGUAGUU-3 (Sense) and 5-CUACCUGAUGGAACGGCACGA GGUU-3 (antisense); HSP70-1: 5-CCAUCUUACG ACUAUUUCUUU-3 (Sense) and 5-AGAAAUAG UCGUAAGAUGGUU-3 (antisense).

### Establishment of MI animal model and drug administration

The Wistar rats used in this study were purchased from Charles River (Beijing China). The animal experiments were approved by the Institutional Animal Care and Use Committee of Fujian Medical University (Approved number: 2018036). These rats were fed on water ad libitum and laboratory diet, and kept in the cage with 35 - 45%, 23 - 26 °C temperature, 12-h light/ dark cycle.

For the RIC treatment experiment, 40 Wistar rats (male, 8-week-old, 280-300 g) were randomly divided into 4 groups, Sham, myocardial infarction (MI), Pre-conditioning (Pre-RIC), and Post-conditioning (Post-RIC). MI was induced by ligating the left coronary artery permanently with a 5-0 polypropylene suture as described previously [27]. Chloral hydrate (10%, 150 mg/kg) was used for anesthesia. Penicillin and morphine were used after surgery to prevent infection and reduce pain. Same procedure was performed for the rats in the group sham excluding the ligation of the left coronary artery. For the group Pre-RIC, the rats were anesthetized adequately, and the bilateral hindlimbs were subjected to RIC treatment (10 cycles of 2 min reperfusion and 2 min bilateral hindlimb ischemia using tourniquets, at 20-second intervals) 2 weeks before MI administration (Once every two days) RIC treatment was performed once every two days. For the group Post-RIC, the animals were subjected to RIC treatment once every two days for 2 weeks after MI administration.

For the RIC exosomes treatment, 18 Wistar rats (male, 8-week-old, 280-300 g) were randomly divided into 2 groups, group control, and group RIC exosomes. MI model was established as described above. Then, the rats in the group RIC exosomes were injected with RIC exosomes through a caudal vein once every three days. The same dose and administration frequency of normal saline was used for group control. The animals were sacrificed at different time points (7, 14, 28 days) for additional experiments.

### Flow cytometry analysis

Flow cytometry analysis was conducted as described previously [28]. 1×10^6^ cells were harvested by centrifuging at 1000 g/min for 5 min within cell stain buffer (Biolegend, 420201). After incubation with the corresponding antibodies for 30 min at 4°C, the reaction was stopped by washing twice with cell stain buffer, and cell apoptosis was measured through flow cytometry.

### Detection of cell cycle

Cells (5×10^4^ each well) were seeded into 6-well plates and cultured 24 h. After culture with RIC exosomes and si-Hsp70 for 48 h, cells were harvested and fixed in 70% ethanol for 4 h. After washing 3 times by PBS, cells were stained by 50 μL PI at 4°C for 1 h. Then, the cell cycle was measured by flow cytometry.

### RNA isolation and real-time PCR

The RNA isolation and real-time PCR were conducted as described previously [29]. TRIzol reagent (Invitrogen, Carlsbad, CA, USA) was used to isolate RNA. 50 ng RNA was reverse-transcribed into cDNA using ReverTra Ace qPCR RT Master Mix with gDNA Remover (TOYOBO). Amplification was conducted for 40 cycles using THUNDERBIRD SYBR qPCR Mix (TOYOBO). The primer information was listed as follows: (1) GAPDH: forward: 5′-ACAACAGCCT CAAGATCATCAG-3′ and reverse: 5′-GGTCCACCA CTGACACGTTG-3′; (2) eNOS: forward: 5′-GGTCAA CTATTCCCTGTCC-3′ and reverse: 5′-ACACCACA TCATACTCATCC-3′; (3) inducible nitric oxide synthase (iNOS): forward: 5′-ATATACCTCCTGAGT GAAG-3′ and reverse: 5′-GGTCCTTGGTTGT AGATA-3′; (4) HIF-1α : forward: 5′-CAGAAGATAC AAGTAGCCTC-3′ and reverse: 5′-CTGCTGGAATAC TGTAACTG-3′; (5) Ang-1: forward: 5′-TATGCCA GAACCCAAAAAGG-3′ and reverse: 5′-GGGCACA TTTGCACATACAG-3′; (6) VEGF: forward: 5′-ATGAACTTTCTGCTGTCTTGGG-3′ and reverse: 5′-CTGTATCAGTCTTTCCTGGTGAG-3′. Relative expression of target genes was calculated using the 2^-ΔΔCt^ method. ΔΔCt=ΔCt experiment - ΔCt control, ΔCt=Ct target gene - Ct control gene. The fold change between the experimental group and the control group=2^-ΔΔCt^.

### Western blot analysis

Western blot analysis was permormed as described [30]. Tissues were lysed in RIPA lysis buffer, and Bradford kit (Bio-Rad, Hercules, USA) was used for measuring protein concentration. Same amount of protein was separated by 8% SDS-PAGE gels and transferred to polyvinylidene fluoride membranes (Merck Millipore, Darmstadt, Germany). The membranes were blocked in Tris-buffered saline containing 5% skim milk for 1 h and cultured with primary antibodies at 4 °C overnight. After washing 3 times with PBS, the membranes were incubated with a secondary antibody (1:1500) at room temperature for 2 h. The protein grey was visualized using the LAS-3000 luminescent image analyzer (Fujifilm, Tokyo, Japan) and analyzed with Quantity One software (Bio-Rad Laboratories, Hercules, USA). The antibodies information was listed as follows: Hsp70 (1:800, ab2787, Abcam, UK); eNOS (1:1000, ab76198, Abcam, UK); iNOS (1:1000, ab49999, Abcam, UK); HIF-1α (1:500, ab6489, Abcam, UK), Ang-1 (1:1500, ab49694, Abcam, UK), and VEGF (1:800, ab9540, Abcam, UK).

### Transwell assay

Cell migration was detected with transwell assay through polycarbonate membrane Boyden chambers in a transwell apparatus (Costar, USA). Cells (2×10^5^ each well) were plated to the up chamber, and 3 mL DMEM containing 15% FBS was used to cover the lower chamber. After 48 h, the lower chamber was fixed with 4% polyformaldehyde for 30 min. After washing 3 times, the lower chamber was stained by Giemsa for 20 min. Then the migrated cells in the three fields were counted with an inverted microscope (Olympus CKX31, Japan).

### Hemodynamic assessment

Hemodynamic assessment of left ventricular function was conducted through transthoracic echocardiography with a Xario ultrasound device (Toshiba Medical Systems, Tokyo, Japan). Briefly, rats were anesthetized with chloral hydrate, and the cannulation was done to the left ventricle. Biofunction experiment system MP100-CE (BIOPAC Systems, Santa Barbara, USA) was used to record hemodynamic parameters. After the hemodynamic assessment, the heart tissues were excised for histopathological analysis.

### Histopathological analysis

After the sacrifice of rats, the heart tissues were collected and fixed in 4% paraformaldehyde for 24 h. Paraffin was used for tissue embedding, and 8-μm thick sections were cut from each segment and stained with Masson trichrome and hematoxylin-eosin (HE). Three slides were chosen for each specimen, and three views were selected for each slide. Zeiss AxioVision (Jena, Germany) was used for capturing.

### Immunohistochemistry staining

The immunohistochemistry staining was performed as described previously [31]. Then heart tissue sections were de-paraffin firstly. Then, sections were successively treated by microwave heating for antigen repair, tissue washing (3 times, 3 min/time), and incubation with 3% H_2_O_2_ (10 min). After washing 2 times (5 min/time), 5% goat serum was used for blocking. Then the primary antibody (vWF, 1:40, DAKO) was applied to incubate tissue overnight at 4°C. After washing, the tissues were incubated with secondary antibody for 2 h at room temperature. Then DAB regent was used to incubate tissues, and an inverted microscope (Olympus CKX31, Japan) was used for capturing.

### Transmission electron microscope (TEM)

The prepared RIC exosomes were identified by TEM. Briefly, the prepared exosomes were suspended in PBS, and placed on the heated and carbon-coated copper grids. Then the grids were then placed on a drop of 2% glutaraldehyde for 10 min. The morphology of exosomes was investigated using HT 7700 TEM (Hitachi, Tokyo, Japan).

### Tube formation

Tube formation assay was used to assess angiogenesis. 100 μL Matrigel (BD Biosciences, USA) was added into μ-Plate Angiogenesis 96-well to form a solid structure. Then the 150 μL suspended cells (2 × 10^6^/mL) with DMEM were plated into wells, and cultured for 24 h at 37°C. Then, tube formation was observed under a light-field microscope. The number of tube branches was counted in three fields.

### Cell proliferation

CCK-8 assay (Nanjing Jiancheng, Nanjing, China) was used to measure cell proliferation. Briefly, cells were plated at 5×10^3^/well into 6-well-plate and incubated for 24 h. After administration with RIC exosomes and Hsp70 siRNA for 24 h, 10 μL reagent was added. After incubation for 2 h, the optical density (450 nm) was measured.

### Statistical analysis

Results are shown as the mean ± standard deviation (SD). Statistical analysis was calculated using SPSS 19.0 (SPSS Co., Ltd., USA). Student's t-test was applied to analyze the statistical significance of the difference between the two groups. P < 0.05 means statistically significant.
